# Melanoma in Primary Care: A Narrative Review of Training Interventions and the Role of Telemedicine in Medical Education

**DOI:** 10.3390/curroncol32090522

**Published:** 2025-09-18

**Authors:** Ignazio Stanganelli, Edoardo Mora, Debora Cantagalli, Serena Magi, Laura Mazzoni, Matelda Medri, Cesare Massone, Davide Melandri, Federica Zamagni, Ines Zanna, Gianluca Pistore, Saverio Caini, Salvatore Amato, Vincenzo De Giorgi, Pietro Quaglino, Maria Antonietta Pizzichetta, Giovanni Luigi Tripepi, Giorgia Ravaglia, Sofia Spagnolini

**Affiliations:** 1 Dermatology Resident Training Program, Department of Medicine and Surgery, University of Parma, 43121 Parma, Italy; ignazio.stanganelli@irst.emr.it (I.S.); giorgia.ravaglia@unipr.it (G.R.); 2 Skin Cancer Unit, IRCCS Istituto Romagnolo per lo Studio dei Tumori “Dino Amadori”-IRST Meldola (FC), 47014 Meldola, Italy; serena.magi@irst.emr.it (S.M.); laura.mazzoni@irst.emr.it (L.M.); matelda.medri@irst.emr.it (M.M.); 3 Dermatology Unit, Ospedale S. Anna, 22042 Como, Italy; 4 Primary Care Resident Training Program, Ausl Bologna, 40121 Bologna, Italy; 5 Dermatology Unit, Ospedali Galliera, 16128 Genova, Italy; cesare.massone@galliera.it; 6 Department of Medical and Surgical Sciences, University of Bologna, 40126 Bologna, Italy; davide.melandri2@unibo.it; 7 Registro Tumori della Regione Emilia Romagna, IRCCS Istituto Romagnolo per lo Studio dei Tumori “Dino Amadori”-IRST Meldola (FC), 47014 Meldola, Italy; federica.zamagni@irst.emr.it; 8 Clinical Epidemiology Unit, Institute for Cancer Research, Prevention and Clinical Network (ISPRO), 53100 Siena, Italy; i.zanna@ispo.toscana.it (I.Z.); s.caini@ispro.toscana.it (S.C.); 9 Clinical Epidemiology Unit, Istituto Dermopatico dell’Immacolata-IRCCS, 00167 Rome, Italy; 10 Associazione Dermatologi Ospedalieri Italiani (ADOI), Sicilia and Teledermatology Working Group, 90146 Palermo, Italy; amatosal1@virgilio.it; 11 Dermatology, Department of Health Sciences, University of Florence, 50121 Florence, Italy; vincenzo.degiorgi@unifi.it; 12 Dermatologic Clinic, Department of Medical Sciences, University of Turin, 10124 Turin, Italy; pietro.quaglino@unito.it; 13 Department of Dermatology, University of Trieste, 34127 Trieste, Italy; pizzichetta@cro.it; 14 Department of Medical Oncology, Centro di Riferimento Oncologico di Aviano (CRO) IRCCS, 33081 Aviano, Italy; 15 Institute of Clinical Physiology, National Research Council CNR-IFC, 89124 Reggio di Calabria, Italy; giovanniluigi.tripepi@cnr.it

**Keywords:** primary care, medical education, melanoma, e-learning, telemedicine

## Abstract

General practitioners play a crucial role in the early detection and prevention of cutaneous melanoma. However, structured training on skin cancer diagnosis and management is often lacking. This narrative review aims to map the current educational interventions for general practitioners focused on melanoma, assess their methodological approaches and outcomes, and explore the contribution of e-learning and telemedicine in medical education. A comprehensive literature search identified 54 relevant studies published between 1 January 1995 and 31 December 2024. Data were extracted and categorized by topics covered, training methodology, interactivity, and clinical outcomes. Training programs varied widely in duration, delivery, and content. Interventions that integrated dermoscopy and interactive methodologies demonstrated improved diagnostic accuracy and clinical impact. E-learning, particularly asynchronous models, emerged as a flexible and effective modality, although few studies evaluated long-term retention or clinical practice changes. Educational programs tailored to general practitioners and enriched with dermoscopy and telemedicine tools show promise in improving melanoma detection and care. Structured, interactive, and blended/hybrid learning models should be prioritized to support effective primary and secondary prevention.

## Simple Summary

General practitioners are often the first to assess suspicious skin lesions, yet many lack specific training in melanoma detection. This narrative review analyzes 54 studies (1995–2024), comparing in-person and e-learning educational interventions in terms of structure, content, duration, interactivity, and diagnostic outcomes. A key finding is that programs incorporating dermoscopy and interactive elements—particularly asynchronous online formats—can enhance sensitivity, specificity, and diagnostic accuracy. However, few studies evaluated long-term clinical impact. Our review provides novel insights into the strengths and limitations of different training approaches and highlights the potential of hybrid models to improve early melanoma recognition in primary care. Structured and accessible training for general practitioners is essential to ensure timely diagnosis, better patient outcomes, and integration into dermatological care pathways.

## 1. Introduction

Proper triage of skin lesions and knowledge of the diagnostic–therapeutic pathway for melanoma enable effective collaboration between general practitioners (GPs) and specialized facilities. Training GPs and pediatricians is a strategic approach for first triage and for promoting both primary and secondary prevention. Primary prevention reduces the incidence of malignant skin tumors by addressing risk factors, while secondary prevention ensures timely diagnosis [1].

Public awareness initiatives for skin self-surveillance have been implemented in many countries and, despite being fragmented, have improved time to diagnosis and survival rates for cutaneous melanoma [1]. However, many GPs still lack adequate training in diagnostics, referral, and management of melanoma. 

Recent systematic reviews identified numerous educational interventions for GPs, ranging from brief online modules to extended courses [2,3]. While several improved melanoma-related knowledge and competence—especially when dermoscopy was incorporated—few evaluated effects on clinical practice through audits or referral analysis [2,3]. This gap highlights the need for more effective and practice-oriented training strategies. This review evaluates the effectiveness of educational interventions for GPs in diagnosing pigmented lesions and melanoma, with particular attention to the pedagogical models applied. It compares traditional in-person formats with synchronous and asynchronous e-learning, analyzing content delivery, learner engagement, assessment methods, and clinical outcomes. By integrating findings from systematic reviews and original studies, it aims to clarify how digital and hybrid education can enhance early melanoma detection and inform future dermatology training programs.

## 2. Materials and Methods

We began our literature search with two systematic reviews conducted by Brown [2] and Gonna [3], which included 51 studies published between 1 January 1995 and 31 December 2020 and 21 studies published between 1 January 2000 and 31 December 2021, respectively. Both reviews focused on skin cancer education programs for primary care physicians. From these, we extracted all relevant studies that specifically addressed melanoma and the early diagnosis of skin cancer in general practitioners (GPs).

Inclusion criteria encompassed studies describing educational interventions specifically targeting GPs or GP residents, with a focus on melanoma and the early diagnosis of skin cancer. Eligible studies included training programs delivered through face-to-face sessions, literature-based formats, e-learning modules, or blended and interactive methods, including, but not limited to, the use of dermoscopy and telemedicine tools.

We excluded studies that did not involve GPs or GP trainees (e.g., those focused on dermatologists or medical students), as well as those that failed to describe the content of the training or did not include any form of outcome measurement or statistical analysis.

Duplicates were removed using reference management software, and ten additional articles were excluded after full-text review because they did not focus exclusively on primary care settings or did not address early skin cancer diagnosis.

To expand and update the evidence base, we conducted a supplementary systematic search of major medical databases: PubMed/MEDLINE, Scopus, Web of Science, and Embase. The search covered publications between 1 January 2020 and 31 December 2024, using the following combination of MeSH terms and free-text keywords: “primary care physicians,” “primary care providers,” “general practitioners,” “family medicine,” “general provider”, “family doctor”, “family physician”, “skin cancer,” “melanoma,” “skin cancer,” “early diagnosis,” “education,” “training,” “educational training,” “e-learning,” “teledermatology,” “dermoscopy,” and “systematic literature review”.

The same inclusion and exclusion criteria applied in the initial selection were adopted for this extended search, and all potentially eligible articles were re-read in full to confirm their suitability.

In addition, five further articles were identified through citation tracking.

**Figure 1:**
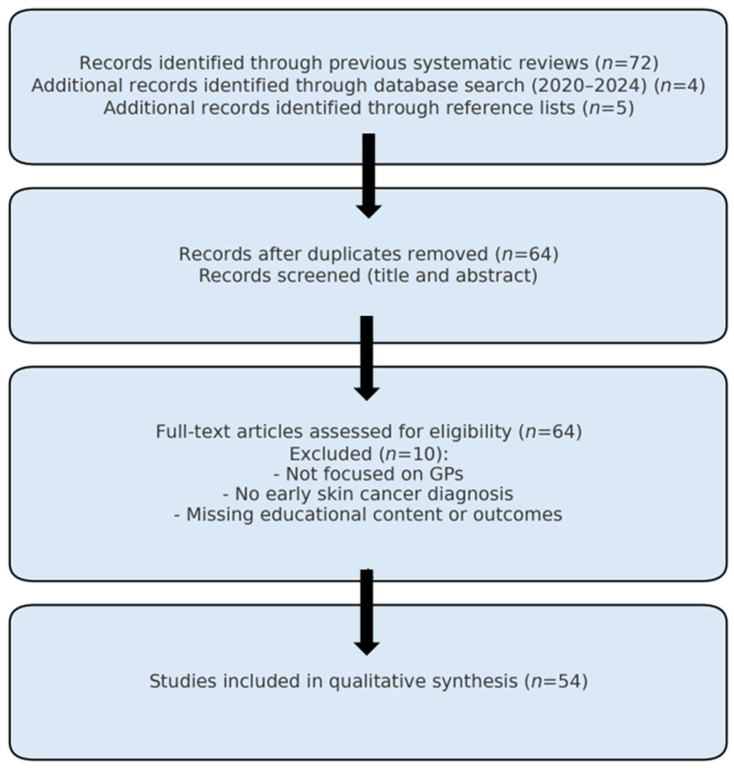
Flowchart. A comprehensive literature search was conducted using two previously published systematic reviews as a starting point, identifying a total of 72 studies [2,3]. After removing duplicates and screening titles and abstracts, 10 articles were excluded for not meeting the inclusion criteria (i.e., lacking focus on general practitioners or early skin cancer diagnosis). An updated search of major medical databases (2020–2024) yielded four additional studies. Reference list screening identified five more articles. Inclusion criteria comprised studies describing educational interventions for general practitioners (GPs) or GP residents, with an emphasis on melanoma or early skin cancer detection, using face-to-face, e-learning, literature-based, or interactive formats. Studies were excluded if they did not involve GPs, lacked description of educational content, or did not report outcomes. In total, 54 studies were included in the review. Data were extracted and organized into thematic domains, including training modality, content, duration, and educational outcomes.

In total, 54 studies met the final eligibility criteria and were included in this narrative synthesis (Figure 1). Of these, 32 used face-to-face formats and 22 adopted e-learning approaches.

Data were extracted and systematically organized into thematic tables summarizing key findings in the following areas:

- Educational interventions and topics addressed;
- Training methodologies (e.g., delivery format, in-person, e-learning, literature-based feedback strategies, patient interaction, interactivity and course duration);
- Outcomes of face-to-face training programs;
- Outcomes of e-learning-based training programs.

## 3. Results

The intervention studies consistently included training on the clinical principles of dermatological diagnosis of melanoma—particularly concerning the ABCDE rule and the “ugly duckling” sign—occasionally supplemented by dermoscopic training. The following table illustrates the key areas addressed in the training courses (Table 1).

### 3.1. Educational Interventions and Topics Addressed

Most of the analyzed studies focused their training programs on selected thematic areas of melanocytic pathology, often without comprehensively addressing the full scope of the integrated care pathway—knowledge that is essential for effective and optimal melanoma management (Table 2). However, a subset of studies [4,5,6,7,8,9,10,11,12,13,14] stand out for offering broader and more integrated curricula. These programs included not only diagnostic criteria for benign and malignant melanocytic lesions but also in-depth training in epidemiology and melanoma care management.

Among the most comprehensive and complex educational initiatives, few studies [13,14,26,55] covered all core components of melanoma training, including: epidemiology, diagnosis of pigmented and non-pigmented skin lesions, management strategies, patient counseling, fundamentals of dermoscopy, and the implementation of diagnostic algorithms.

Within the context of these numerous studies and their heterogeneous scope, a subset included training interventions featuring sessions on basic dermoscopy concepts [5,7,15,16,28,32,36,38,42,50]. The introduction of dermoscopy into primary care practice has shown substantial advantages, including:

1. A reduction in the number of benign lesions unnecessarily referred to dermatologists;
2. An improved benign-to-malignant ratio among excised lesions;
3. Measurable improvements in the diagnostic accuracy and clinical decision-making of trained GPs;
4. Demonstrated effectiveness even among practitioners with limited prior experience, especially when reinforced by refresher courses;
5. A favorable cost-effectiveness profile, with positive implications for both healthcare systems and patient mobility.

### 3.2. Training Delivery Methods and Implementation Strategies

Over the past thirty years, numerous training initiatives have been developed for GPs, employing a wide range of delivery formats. Table 3 summarizes the key characteristics of these interventions, including the type of delivery (synchronous or asynchronous), presence of feedback mechanisms, level of interactivity, integration of supporting literature, opportunities for patient interaction, and overall course duration.

An analysis of the methodologies employed in the selected studies, as shown in Table 3, reveals that live (synchronous) sessions represent the most adopted training modality, featured in 32 studies. This preference likely reflects the accessibility of live sessions, particularly for practitioners with limited familiarity or confidence in using newer digital technologies. A significant number of studies employ more than one approach to complete their training programs, and in some cases, they include direct references to the relevant scientific literature [8,10,11,12,16,17,19,22,25,28,29,30,32,39,40,41,45,50,56].

Moreover, e-learning has been adopted in 22 of the studies examined, emerging as a training modality increasingly used in recent years thanks to the rapid spread of new digital technologies and the undeniable advantages of interactivity and personalization in the learning experience [10,11,13,14,21,24,26,28,29,30,32,33,38,39,41,45,46,47,51,53,55,60].

The use of interactivity, enabled by specific content such as quizzes, multiple- or single-choice questions, tests, and simulations—thereby stimulating and promoting an active learning process—has been employed in several training programs [5,6,7,10,11,24,26,32,33,35,39,40,41,45,46,47,51,53,54,55].

Feedback strategies designed for participants were incorporated into several training interventions [5,7,29,30,32,43,44,45,46,47,57]. Given that feedback can confirm and restructure previously acquired information, thereby reinforcing and consolidating the learning process, its use constitutes a powerful strategy for enhancing participants’ knowledge and skills. However, it should be noted that only a limited number of studies [11,26,28,33] included feedback questions providing information on user satisfaction with the platform as well as suggestions for its improvement.

Only five studies incorporated direct patient interaction during the training intervention, specifically through sessions focused on evaluating pigmented or non-pigmented skin lesions, with or without the aid of dermoscopy [4,20,31,32,45]. 

The duration of the training programs varied considerably, ranging from a single day (with a variable number of instructional hours) to multiple days of participation in several training sessions.

#### 3.2.1. Frontal Learning Courses: Comparison of Results

Frontal learning, a traditional face-to-face educational approach, was employed in several studies included in this analysis to train GPs in the clinical diagnosis of dermatological conditions. Table 4 provides a structured summary of the included studies, detailing the study design number of participants, types of interventions, and key diagnostic outcomes (sensitivity, specificity, accuracy, and positive and negative predictive values). It focuses on face-to-face training interventions and includes only those studies that reported data on the number of participants before and after the training, along with measures of intervention effectiveness.

Out of a total of 5903 GPs contacted, 2020 completed the pre-training questionnaire, while 1442 completed the post-training questionnaire. Among the groups of GPs who participated in both assessments, varying proportions were trained using frontal education modalities, depending on the study. The effectiveness of these training programs was evaluated by monitoring indicators reflecting participants’ diagnostic performance before and after the intervention.

Across all the studies that assessed it, a consistent increase in diagnostic sensitivity was observed following the training, although this improvement was not statistically significant in every case. Regarding specificity and overall diagnostic accuracy, values remained stable in four out of the eight studies for which data were available, while three studies reported improvements in both parameters.

Several studies provided clear evidence of effectiveness. For instance, Bedlow et al. reported increases in sensitivity from 63% to 76% and specificity from 55% to 62%, and accuracy remained stable [17]. Brochez et al. demonstrated a broad improvement across all diagnostic parameters, including sensitivity (72% to 84%), specificity (71% to 70%), accuracy (49% to 56%), positive predictive value (61–63%), and negative predictive value (80–87%) [8]. Similar improvements were noted by Argenziano et al., who observed a significant increase in sensitivity (from 54.1% to 79.2%) and a slight gain in specificity (from 71.3% to 71.8%), with a high negative predictive value of 95.8% [15]. Meanwhile, Seiverling et al. reported sensitivity gains from 62,5% to 88,1%, specificity from 90.3% to 87.8%, and accuracy from 59.7% to 78.3% [51].

Some studies [36,44,56] reported only partial outcomes or focused on knowledge-based assessments without fully presenting diagnostic metrics. Others [12,25,27] lacked post-test results or key performance indicators, limiting the ability to evaluate impact. The studies by Harkemanne et al. [34,61] introduced simulated scenarios and longitudinal follow-up but did not include complete statistical data.

#### 3.2.2. E-Learning Courses: Comparison of Results

E-learning, a digital and remote educational approach, was utilized in several of the studies included in this analysis to train GPs in the clinical diagnosis of dermatological conditions. We can distinguish three main e-learning training modalities based on the degree of synchronism and flexibility for participants (Table 5):

1. Synchronous Training: This modality involves real-time interaction between participants and instructors through platforms like webinars and virtual classrooms. It enables immediate feedback but requires precise scheduling and coordination.
2. Asynchronous Training: Participants learn independently at their own pace, accessing materials on demand. This flexible format promotes self-directed learning but lacks real-time interaction and immediate instructor feedback.
3. Blended Learning: Combining synchronous and asynchronous elements, this approach balances flexibility with engagement. It requires careful planning to allocate content appropriately between self-paced and live sessions, optimizing interaction and knowledge transfer.

In this review, we distinguish between two related but distinct educational modalities:

1. Blended learning: refers to a combination of synchronous and asynchronous e-learning activities conducted entirely online.
2. Hybrid learning models: combine digital (online) components with in-person (face-to-face) sessions, such as workshops or clinical case discussions.

While both approaches aim to enhance flexibility and engagement, hybrid models allow for direct interpersonal interaction, which may support the development of practical skills in clinical settings. The effectiveness of these training programs was evaluated by monitoring indicators reflecting participants’ diagnostic performance before and after the intervention.

Across all the studies that assessed it, a consistent increase in diagnostic sensitivity was observed following the training, although this improvement was not statistically significant in every case. Regarding specificity, values improved after training in all the records where this data was available. Overall diagnostic accuracy improved in five out of five studies in which data were available. These findings indicate that e-learning interventions tend to improve sensitivity, specificity, and overall diagnostic accuracy. Moreover, this modality can reach a very large audience, making it an excellent training approach.

Table 6 provides a structured summary of the included studies, outlining the study design, sample size, types of interventions, and key diagnostic outcomes (sensitivity, specificity, accuracy, positive and negative predictive values). It focuses on e-learning formats and presents a comparative overview of 16 studies evaluating the effectiveness of e-learning interventions in improving general practitioners’ diagnostic performance for skin cancer.

Out of a total of 12,284 general practitioners (GPs) contacted, 2579 participants completed the pre-training questionnaire, while 2185 completed the post-training questionnaire.

The included studies varied considerably in sample size, geographic origin, methodology, and outcome reporting. Most studies demonstrated improvements in diagnostic knowledge or accuracy following the intervention. For instance, Gerbert et al. reported an increase in diagnostic accuracy from 43% to 56% [30], while Harris et al. documented improvements in sensitivity (from 91% to 95%), specificity (from 72% to 92%), and overall accuracy (from 52% to 85%) [11]. Eide et al. showed modest gains in sensitivity, specificity, and accuracy, sustained up to six months post-intervention [26]. Nervil et al. also reported improvements in sensitivity (from 67.1% to 73.7%) and diagnostic accuracy (from 42.5% to 53%) [43]. Only a limited number of studies provided complete diagnostic metrics, including sensitivity (Se), specificity (Sp), accuracy, positive predictive value (PPV), and negative predictive value (NPV). Menzies et al. was the only study to report all five parameters, showing significant improvements: sensitivity increased from 23.1% to 69.2%, specificity from 90.6% to 92.8%, PPV from 18.8% to 47.4%, and NPV from 93.3% to 97.9% [39].

Some studies, such as Dolianitis et al. and Pagnanelli et al., focused on comparisons between diagnostic algorithms or dermoscopic criteria rather than using pre- and post-test designs [24,59]. Others, like Secker et al. and Robinson et al., assessed diagnostic ability across specific lesion categories [46,50]. Notably, large-scale interventions by Markova et al. and Weinstock et al. involved thousands of patients, highlighting the potential for broad clinical implementation [37,55].

Among the studies analyzed, four employed a hybrid educational model combining both live (face-to-face) and e-learning components: Gerbert et al., Menzies et al., Secker et al., and Marra et al. [30,38,39,50] These hybrid studies illustrate the flexibility of combined learning approaches in GPs education. Menzies et al. reported the most comprehensive diagnostic outcome set, with substantial improvements across all metrics [39]. Marra et al. found a post-training diagnostic accuracy of 70.3%, despite not reporting sensitivity or specificity values [38]. Gerbert et al. documented modest gains in diagnostic accuracy (from 43% to 56%) [30], while Secker et al. provided data stratified by lesion type [50]. However, the lack of standardized and complete outcome reporting in several of these studies limits direct comparability.

## 4. Discussion

Training is essential to maintain high-quality patient care, as medical education must evolve with emerging evidence and advancing clinical practices. Literature shows that various training programs have been developed to improve GPs’ skills in diagnosing skin cancers, especially melanoma. However, only a small number of these interventions have led to positive changes in clinical practice (i.e., statistically significant improvements) [32,38].

Due to the lack of standardization in how these training activities are organized, it is difficult to compare the outcomes obtained. Indeed, multiple parameters can be considered when assessing the effectiveness of a training program, such as increased theoretical knowledge, greater confidence in previously acquired information, and expanded skill sets. Other crucial aspects include enhanced self-efficacy and, more specifically, the training’s impact on diagnostic skills in terms of sensitivity, specificity, and negative and positive predictive values.

Our review showed that over the past thirty years only a few studies have methodically focused on a detailed analysis of these parameters. Although most studies report improvements in at least one key outcome parameter (knowledge, skills, self-efficacy, or diagnostic performance), some studies demonstrate limited gains in knowledge and/or clinical practice [20,21,23,27,44]. It has been hypothesized that training interventions lacking active interaction and of short duration are unlikely to produce clear and lasting changes in clinical practice. By contrast, courses on dermoscopy designed for GPs have proven effective at increasing the ability to detect cutaneous melanomas, outperforming traditional naked-eye examinations [62]. Supporting this premise, several cited studies that included a dermoscopy component showed a statistically significant increase in participants’ skills [24,47,56]. In the study by Menzies et al. [39], improvements were observed in self-efficacy and diagnostic performance, as well as positive changes in clinical practice. Furthermore, our review highlights substantial variability in the content of training programs on skin cancer in primary care. There is no standardized training framework across studies; the choice of topics is highly variable. Only a few programs provide a holistic educational approach, combining clinical content with practical tools and communication skills. More recent interventions tend to be more comprehensive, possibly due to advances in dermoscopic techniques and a better understanding of educational needs in primary care. A few studies offered comprehensive, integrated curricula. In contrast, other programs focused narrowly on lesion recognition [13,14,37,41,53,55].

Recent reviews [61,63,64,65,66,67] provide foundational insights into the role of educational interventions—particularly e-learning—in improving GPs’ ability to diagnose skin cancers. However, when compared with the broader dataset captured in the tables of this review, some important differences and innovations emerge.

E-learning, as highlighted in both reviews, offers clear advantages in terms of flexibility, asynchronous learning, and the ability to scale across large groups of practitioners. Posada et al. emphasize the use of interactive online modules, often incorporating dermoscopy, and report improved diagnostic performance in knowledge, accuracy, and confidence [67]. Harkemanne et al., while confirming the presence of e-learning components, did not quantify their prevalence and reported a high degree of heterogeneity in methods and content [61].

Table 4 and Table 6 analyzed in this narrative review significantly expand upon these findings, identifying 16 e-learning studies and 20 frontal learning studies, many of which were not included in the previous reviews. This allowed for a more granular comparison of effectiveness based on pre-/post-test outcomes, sensitivity, specificity, and long-term retention (e.g., Eide et al. with 6-month follow-up [26]). The additional studies also revealed a wider variety of formats, including hybrid models, which blend online modules with face-to-face dermoscopy workshops or clinical case discussions.

In contrast, frontal (in-person) learning programs demonstrated strengths in practical skill development, especially in the application of dermoscopy and diagnostic algorithms. Several of these interventions [8,15,51] showed substantial gains in diagnostic accuracy, often supported by structured evaluation tools and simulated clinical scenarios, highlighting the unique value of hands-on instruction in fostering clinical decision-making and confidence. These findings suggest that hybrid models may offer effective alternatives to purely digital or purely in-person formats, but further research with standardized evaluation frameworks is needed to validate their impact.

Moreover, the extended dataset allowed the identification of new thematic trends not captured in the original reviews. Recent studies [13,14] incorporated fully integrated curricula that addressed not only lesion recognition but also epidemiology, management, counseling, and the use of algorithms, offering a more holistic approach to primary care training in skin cancer. This contrasts with the more limited thematic scope of many earlier interventions, which focused primarily on lesion identification.

In the new digital era, embedding AI into telemedicine education represents a forward-looking strategy to enhance primary care capacity for melanoma detection and management. Artificial intelligence (AI) and mobile health applications in medical education can provide future practitioners with practical experience in combining decision-support algorithms with clinical judgment. Two recent systematic reviews have comprehensively evaluated the current state of AI in dermatology. A meta-analysis by Salinas et al. [68] reported that AI achieved a sensitivity of 87.0% and a specificity of 77.1%, comparable or superior to clinicians (79.8% and 73.6%, respectively) and similar to experienced dermatologists. These findings suggest that AI could serve as a valuable second-opinion tool, particularly for non-specialists or in areas with limited access to dermatology services.

Similarly, a recent systematic review by Zararsis et al. [60] confirmed these promising findings but highlighted the lack of real-world validation and the limited integration of AI tools into primary care workflows. Despite the rapid evolution of this field, both reviews emphasized that AI systems remain largely confined to research and proof-of-concept phases. Real-world validation, regulatory approval, and practical integration into existing educational and diagnostic pathways are still limited. Further studies are required to assess their impact in everyday clinical practice.

This narrative review has several limitations. The high methodological heterogeneity among studies—in design, training methods, and outcome measures—precluded meta-analysis and limited direct comparisons. Most studies were observational or pilot programs, with few randomized controlled trials, weakening overall evidence quality. Outcome reporting was inconsistent, reducing comparability. While the search was broad and up to date, no formal risk of bias assessment was conducted, and selection bias cannot be ruled out. Language and database restrictions may have excluded relevant studies despite multi-database searching and citation tracking. Nevertheless, this review offers a timely synthesis of educational strategies for melanoma detection in general practice, identifying key strengths and areas for future improvement.

## 5. Conclusions

Studies in the literature indicate that a wide range of educational initiatives have been developed to train GPs in the primary and secondary prevention of cutaneous melanoma. The greatest challenge highlighted in most of these studies is converting gains in knowledge and self-efficacy into meaningful changes in clinical practice and, ultimately, patient care. An effective training program focused on skin cancers—particularly melanoma—and designed for primary care physicians can help reduce morbidity and mortality, especially where access to dermatological services is limited. Both e-learning and in-person educational programs have demonstrated effectiveness in improving GPs’ diagnostic skills in skin cancer. However, the absence of standardized outcome measures and limited long-term follow-up continue to limit comparability across studies.

Notably, the interventions that achieved tangible improvements in clinical practice were predominantly e-learning courses incorporating interactive methodologies that foster an active, dynamic learning process and enable broad dissemination of content. In contrast, short-term, passive training interventions without interactivity have proven ineffective. Courses that include a dermoscopy component have shown especially strong outcomes, significantly enhancing diagnostic performance. Dermoscopy training is becoming increasingly prevalent in skin cancer education, given its higher diagnostic sensitivity compared to traditional naked-eye examination and its applicability in primary care settings. AI in dermatology shows promising accuracy comparable to dermatologists and could support melanoma detection in primary care. However, its role remains experimental, requiring real-world validation and integration into clinical practice.

Taken together, these findings from the literature are particularly valuable in shaping the structure and key features of GP-oriented training interventions, making them more likely to yield sustained benefits in clinical practice [3,67,69,70]. While e-learning ensures accessibility, consistency, and scalability, frontal learning remains essential for developing hands-on diagnostic competence. The combination of both, particularly in hybrid models, may represent the most effective strategy. Broader literature reveals a trend toward more comprehensive, multidisciplinary training programs, emphasizing the need for standardized content, validated evaluation metrics, and long-term follow-up in future educational research.

## Figures and Tables

**Table 1:** Topics of training courses.

*Epidemiology*	Melanoma Incidence and Mortality Trends Risk Factors
***Pigmented skin lesions***	Basic principles for the recognition of melanoma and benign pigmented lesions
***Non pigmented skin lesions***	Basic principles for the recognition of squamous cell carcinoma, basal cell carcinoma and benign non-pigmented lesions
***Dermoscopy***	Basics of dermoscopy aimed at recognizing suspicious lesions for skin cancers, in particular melanoma
***Algorithm***	Teaching of a clinical and/or dermatoscopic algorithm (new or pre-existing) to aid the identification of suspicious skin lesions
***Management***	In-depth study of the integrated care pathway (ICP) of melanoma and skin cancers
***Counseling***	In-depth study of prevention strategies such as photoprotection and self-examination of the skin

The terms in the first column, written in bold and italic, stand for represent the main topics covered in the training courses, while the text in the right-hand column provides the detailed content of each topic.

**Table 2:** Articles present in the global literature: topics included in the training intervention.

Author	Epidemiology	Pigmented Skin Lesions	Non Pigmented Skin Lesions	Dermoscopy	Algorithm	Management	Counseling
*Anders et al.* *(2017) * *[4]*	X	X	X			X	X
*Argenziano et al. (2006) * *[15]*		X	X	X	X		
*Augustsson et al. (2019) * *[16]*		X	X	X	X		
*Badertscher* *et al. (2011) * *[5]*	X	X	X	X		X	
*Badertscher* *et al. (2013) * *[6]*	X	X	X	X		X	
*Badertscher* *et al. (2015) * *[7]*	X	X	X	X		X	
*Bedlow et al.* *(2001) * *[17]*		X	X				
*Beecher et al.* *(2018) * *[18]*		X	X			X	
*Bourne et al.* *(2012) * *[19]*		X	X	X	X		
*Brochez et al.* *(2001) * *[8]*	X	X				X	
*Burton et al.* *(1998) * *[20]*	X	X				X	
*Carli et al.* *(2005) * *[9]*	ꓫ	ꓫ				ꓫ	ꓫ
*De Gannes et* *al. (2004) * *[21]*	X	X	X			X	X
*Del Mar et al.* *(1995) * *[22]*		X			X	X	
*Dolan et al.* *(1997) * *[23]*	X	X	X				X
*Dolianitis et al. (2005) * *[24]*		X		X	X		
*Duarte et al.* *(2018) * *[25]*	ꓫ	ꓫ	ꓫ		ꓫ		
*Eide et al.* *(2012) * *[26]*	X	X	X	X	X	X	X
*English et al.* *(2003) * *[27]*		X			X	X	
*Friche et al.* *(2024) * *[28]*		X	X	X			
*Gerbert et al.* *(1998) * *[29]*	X	X	X			X	X
*Gerbert et al.* *(2002) * *[30]*	X	X	X			X	X
*Girgis et al.* *(1995) * *[31]*	X	X				X	
*Grange et al.* *(2014) * *[10]*	X	X			X	X	
*Grimaldi et* *al. (2009) * *[32]*		X	X	X	X		
*Gulati et al.* *(2015) * *[33]*		X	X			X	
*Harkemanne et al. (2020) * *[34]*	X	X			X		
*Harris et al. (1999) * *[11]* *Harris et al.* *(2001) * *[35]*	X	X			X	X	X
*Koelink et al.* *(2014) * *[36]*	X	X	X	X	X		
*Markova et al. (2013) * *[37]*	X	X	X		X	X	X
*Marra et al.* *(2021) * *[38]*		X	X	X		X	X
*Menzies et al.* *(2009) * *[39]*		X		X			
*Mikkilineni et al. (2001) * *[40]*	X	X	X		X	X	X
*Mikkilineni et al. (2002) * *[41]*	X	X	X		X	X	X
*Moscarella et* *al. (2017) * *[42]*		X	X	X	X	X	
*Nervil et al. (2013) * *[43]*		X	X	X			
*Peuvrel et al.* *(2009) * *[12]*	X	X			X	X	X
*Raasch et al.* *(2000) * *[44]*		X	X			X	
*Rivet et al.* *(2018) * *[45]*	X	X	X			X	X
*Robinson et al. (2018) * *[46]*	X	X		X	X	X	
*Robinson et al. (2018) * *[47]*	X	X		X	X	X	
*Rogers et al.* *(2016) * *[48]*				X	X		
*Sawyers et al.* *(2020) * *[49]*				X	X		
*Secker et al.* *(2017) * *[50]*		ꓫ		ꓫ		ꓫ	
*Seiverling et al. (2019) * *[51]*		X	X	X	X		
*Shariff et al.* *(2010) * *[52]*		X	X				
*Swetter et al.* *(2017) * *[53]*	X	X	X		X	X	X
*Ward et al.* *(1995) * *[54]*	X	X	X			X	
*Weinstock et* *al. (2016) * *[55]*	X	X	X	X	X	X	X
*Westerhoff et* *al. (2000) * *[56]*		X		X	X		
*Youl et al.* *(2007) * *[57]*	X	X		X	X	X	
*Stanganelli et al. * *(2024), Zamagni et al.* *(2024) * *[13,14]*	X	X	X	X	X	X	X
*De Bedout et al. (2021) * *[58]*		X	X	X	X		
*Pagnanelli et al. (2003) * *[59]*		X	X	X	X		

Bold terms indicate overarching themes synthesized for each article, while italicized text in the first column details each article included in the table.

**Table 3:** Articles in the global literature: methodology.

Author	Country (N° GPs *)	Live	Literature	E-Learning	Feedback	Interaction with Patient	Interactivity	Modality **	Days ***
*Anders et al. * *[4]*	DE (573)	X						S	N.A
*Argenziano et al. (2006) * *[5]*	IT, ES (73)	ꓫ						S	Sing
*Augustsson et al.* *(2019) * *[6]*	SE (56)	X	X					S	Sing
*Badertscher* *et al. (2011) * *[7]*	CH (60)	X			X		X	S	S.M.
*Badertscher* *et al. (2013) * *[8]*	CH (78)	X			X		X	S	S.M.
*Badertscher* *et al. (2015) * *[9]*	CH (78)	X			X		X	S	S.M.
*Bedlow et al. (2001) * *[10]*	UK (17)	X	X					S	Sing
*Beecher et al.* *(2018) * *[11]*	IE (23)	X						S	Sing
*Bourne et al.* *(2012) * *[12]*	AU (4)	X	X					S	N.S.
*Brochez et al.* *(2001) * *[13]*	BE (160)	X	X					S	Sing
*Burton et al.* *(1998) * *[14]*	AU (143)	X				X		A	S.M.
*Carli et al.* *(2005) * *[15]*	IT (41)	X						S	Sing.
*De Gannes et* *al. (2004) * *[16]*	CA (52)			X				S	Sing.
*Del Mar et al.* *(1995) * *[17]*	AU (93)	X	X					A	S.M.
*Dolan et al.* *(1997) * *[18]*	US (82)	X						S	Sing.
*Dolianitis et al.* *(2005) * *[19]*	AU (35)		X	X			X	A	Sing.
*Duarte et al.* *(2018) * *[20]*	PT (11)	X	X					S	Sing.
*Eide et al.* *(2012) * *[21]*	US (54)			X			X	A	Sing.
*English et al.* *(2003) * *[22]*	AU (468)	X	X					A	S.M.
*Friche et al.* *(2024) * *[23]*	FR (134)		X	X				A	S.M.
*Gerbert et al. (1998) * *[24]*	USA (77)	X	X	X	X		X	A	S.M.
*Gerbert et al.* *(2002) * *[25]*	USA (46)	X	X	X	X		X	A	S.M.
*Girgis et al.* *(1995) * *[26]*	AU (41)	X				X		A	3 S.
*Grange et al.* *(2014) * *[27]*	FR (398)	X	X	X			X	S	Sing.
*Grimaldi et* *al. (2009) * *[28]*	IT (13)	X		X	X	X	X	S	S.M.
*Gulati et al.* *(2015) * *[29]*	UK (967)			X			X	A	S.M.
*Harkemanne et al.* *(2020) * *[30]*	BE (56)	X						S	Sing.
*Robinson (1999)* *Harris et al. (2001) * *[31,32]*	UK (354)		X	X			X	A	S.M.
*Koelink et al.* *(2014) * *[33]*	NL (53)	X						S	S.M.
*Markova et al. (2013) * *[34]*	US (57)			X				S	Sing.
*Marra et al.* *(2021) * *[35]*	NL (185)	X		X				A	S.M.
*Menzies et al.* *(2009) * *[36]*	AU (63)	X	X	X			X	A	S.M.
*Mikkilineni et al. (2001) * *[37]*	US (22)	X	X				X	S	Sing.
*Mikkilineni et al. (2002) * *[38]*	US (28)	X	X				X	S	Sing.
*Moscarella et* *al. (2017) * *[39]*	IT (N.S.)	X	X					N.A.	N.A.
*Nervil et al. (2013) * *[40]*	DK (115)			X	X			A	N.A.
*Peuvrel et al.* *(2009) * *[41]*	FR (210)	X	X					S	Sing.
*Raasch et al. (2000) * *[42]*	AU (46)	X			X			A	S.M.
*Rivet et al.* *(2018) * *[43]*	CA (25)	X	X	X	X	X	X	A	S.M.
*Robinson et al.* *(2018) * *[44]*	US (89)			X	X		X	A	S.M.
*Robinson et al.* *(2018) * *[45]*	US (44)			X	X		X	A	S.M.
*Rogers et al.* *(2016) * *[46]*	US (41)	X						S	Sing.
*Sawyers et al.* *(2020) * *[47]*	CA (33)	X						S	Sing.
*Secker et al.* *(2017) * *[48]*	NL (309)	X	X	X				A	Sing.
*Seiverling et al.* *(2019) * *[49]*	US (59)	X					X	S	Sing.
*Shariff et al.* *(2010) * *[50]*	UK (94)	X	X					A	N.S.
*Swetter et al.* *(2017) * *[51]*	US (6)			X			X	A	Sing.
*Ward et al.* *(1995) * *[52]*	AU (N.S.)	X					X	S	Sing.
*Weinstock et al. (2016) * *[53]*	US (108)			X			X	A	Sing.
*Westerhoff et al. (2000) * *[54]*	AU (74)	X	X					S	Sing.
*Youl et al. (2007) * *[55]*	AU (16)	X			X			A	S.M.
*Stanganelli et al. (2024), Zamagni et al. (2024) * *[56,57]*	IT (298)			X			X	A	N.A.
*De Bedout et al. (2021) * *[58]*	CO (21)	X			X		X	S	S.M.
*Pagnanelli et al. (2003) * *[59]*	IT (16)			X			X	N.A.	N.A.

* GP = general practitioner; ** A = asynchronous, S = synchronous, N.A. = not applicable; *** Sing. = single day N S. = number of sessions (if specified), S.M. = multiple sessions (if not specified), N.S. = not specified. Bold terms indicate overarching themes synthesized for each article, while italicized text in the first column details each article included in the table.

**Table 4:** Summary of face-to-face training programs: study design, number of GPs, intervention characteristics, and diagnostic outcomes.

Article	N° GPs	Pre-Test	Post-Test	Post-Test (2)	Se	Sp	Accuracy	VPP	VPN
*Girgis et al., 1995, CH * *[31]*	59	41	41	-	-	-	-	-	-
*Burton et al., 1998, AU * *[20]*	74	63	63	-	-	-	-	-	-
*Raasch et al., 2000, AU * *[44]*	46	46	46	-	72.2%/ 77.1%	44.7%/ 37.3%	-	-	-
*Westerhoff et al., 2000, UK * *[56]*	74	74	74	-	54.6%/62.7%	-	-	-	-
*Bedlow et al., 2001, UK * *[17]*	23	17	17	-	63%/76%	55%/62%	56%/56%	-	-
*Brochez et al., 2001, BE * *[8]*	1956	160	146	-	72%/84%	71%/70%	49%/56%	61%/ 63%	80%/87%
*English et al., 2003, AU * *[27]*	1221	468	-	-	-	-	-	-	-
*Carli et al., 2005, IT * *[9]*	41	41	41	-	46.8%/76.2%	55%/73.1%		-	-
*Argenziano et al., 2006, IT * *[15]*	88	73	73	-	54.1%/79.2%	71.3%/71.8%	-	-	11.3%/ 95.8%
*Youl et al., 2007, AU * *[57]*	16	16	16	-	62.3%/64.5%	-	-	-	-
*Peuvrel et al., 2009, FR * *[12]*	210	87	87	-	-	-	-	-	-
*Shariff et al., 2010, UK * *[52]*	94	94	-	-	-	-	23.5%/21.2%	-	-
*Badertscher et al., 2015, CH * *[7]*	1000	78	78	74	-	-	63.8%/77.7%/66.6%		-
*Koelink et al., 2014, NL * *[36]*	281	53	53	-	-	-	-	-	-
*Anders et al., 2017, DE * *[4]*	573	573	573	-	-	-	62%/77.2%	-	-
*Duarte et al., 2018, PT * *[25]*	11	-	-	-	-	-	-	-	-
*Seiverling et al., 2019, US * *[51]*	59	59	59	-	62.5%/88.1%	90.3%/87.8%	59.7%/78.3%	-	-
*De Bedout et al., 2021, CO * *[58]*	21	21	19	-	62.9%/86.5%	54.7%/75.7%	-	-	-
*Harkemanne et al., 2020, BE * *[34]*	56	56	56	-	-	-	-	-	-

Bold terms indicate overarching themes synthesized for each article, while italicized text in the first column details each article included in the table.

**Table 5:** Comparison of synchronous, asynchronous and blended training.

	Synchronous Training	Asynchronous Training	Blended Learning
*Simultaneous presence of teachers and students*	X		Combination of synchronous and asynchronous training elements (variable according to the structure of the training project)
*Possibility to ask questions to the teacher*	X	X (*Chat e forum*)	
*Possibility to do exercises*	X (in real time)	X	
*Feedback from faculty and attendees*	X (immediate)	X (*Chat e forum*)	
*Management of individual learning times*		X	
*Possibility to carry out training wherever you prefer*	X	X	
*Ability to train whenever you want*		X	
*Possibility to review the training materials*		X	
*Training defined by a medium-long duration time program (weeks/months/year)*		X	

This table presents a comparison of synchronous, asynchronous, and blended training, with the table entries highlighted in bold. The features of each training modality are described in italics in the first column and summarized in the table.

**Table 6:** Summary of e-learning training programs: study design, number of GPs, intervention characteristics, and diagnostic outcomes.

*Article*	*N° GPs*	*Pre-Test*	*Post-Test*	*Post-Test (2)*	*Se*	*Sp*	*Accuracy*	*VPP*	*VPN*
*Gerbert et al., 1998, US * *[29]* *	77	65	52	-	-	-	43%/56%	-	-
*Harris et al., 2001, UK * *[35]*	691	354	354	-	91%/95%	72%/92%	52%/85%	-	-
*Mikkilineni et al., 2001, US * *[40]*	28	22	22	-	-	-	-	-	-
*Pagnanelli et al., 2003, IT * *[59]*	16	16	16	-	for each different method				
*Dolianitis et al. 2005, AU * *[24]*	61	61	-		comparison of different algorithms				
*Menzies et al., 2009, AU * *[39]* *	102	63	63	-	23.1%/69.2%	90.6%/92.8%	-	18.8%/47.4%	93.3%/97.9%
*Eide et al., 2013, US * *[26]*	54	54	54	54 (6 months)	39.4%/ 48.8%/ 44%	44.8%/56.7%/46%	41.5%/51.9%/44.8%	-	-
*Markova et al., 2013, US * *[37]*	57 GP + 3341 patients	57	57 (1−2 months)	57 (12 months)	-	-	-	-	-
*Gulati et al., 2015, UK * *[33]*	8163	1002	1007	-	-	-	-	-	-
*Weinstock et al., 2016, US * *[55]*	618 GP + 16,472 patients	-	8 months	-	-	-	-	-	-
*Secker et al., 2017, NL * *[50]* *	309	293	293	-	For each category of lesion			-	-
*Robinson et al., 2018, US * *[46,47]*	181	90	89	-	For each category of lesion			-	-
*Marra et al., 2021, NL * *[38]* *	194	-		-	-	-	70.30%	-	-
*Nervil et al., 2023, DK * *[43]*	279	115	115	-	67.1%/73.7%	-	42.5%/53%	-	-
*Friche et al., 2024, FR * *[28]*	134	89	63	-	-	-	-	-	-
*Stanganelli et al (2024), Zamagni et al. (2024) * *[13,14]*	1320	298	-	-	-	-	-	-	-

* Hybrid studies: they employed both live and e-learning methods. Bold terms indicate the topics synthesized for each article, while italicized text in the first column details each article included in the table.

## Data Availability

The raw data supporting the conclusions of this article will be made available by the authors on request.

## Abbreviations

The following abbreviations are used in this manuscript:

GPs	General Practitioners
PPV	Positive Predictive Value
NPV	Negative Predictive Value
